# Bacterial Memory of Persisters: Bacterial Persister Cells Can Retain Their Phenotype for Days or Weeks After Withdrawal From Colony–Biofilm Culture

**DOI:** 10.3389/fmicb.2018.01396

**Published:** 2018-06-26

**Authors:** Saki Miyaue, Erika Suzuki, Yoko Komiyama, Yu Kondo, Miki Morikawa, Sumio Maeda

**Affiliations:** ^1^Graduate School of Humanities and Sciences, Nara Women’s University, Nara, Japan; ^2^Faculty of Human Life and Environment, Nara Women’s University, Nara, Japan

**Keywords:** persister, memory, *Escherichia coli*, colony biofilm, antibiotic

## Abstract

Persister cells, or persisters, are a specific subpopulation of bacterial cells that have acquired temporary antibiotic-resistant phenotypes. In this study, we showed that *Escherichia coli* produces many more persister cells in colony–biofilm culture than in the usual liquid culture and that these persisters can be maintained in higher numbers than those from liquid culture for up to 4 weeks at 37°C in a fresh, nutrient-rich, antibiotic-containing medium, even after complete withdrawal from the colony–biofilm culture. This suggests the presence of a long-retention effect, or “memory effect”, in the persister cell state of *E. coli* cells. We also discovered that such increases in persisters during colony–biofilm culture and their memory effects are common, to a greater or lesser degree, in other bacterial species. This is true not only for gram-negative bacteria (*Acinetobacter* and *Salmonella*) but also for gram-positive bacteria (*Staphylococcus* and *Bacillus*). This is the first report to suggest the presence of a common memory mechanism for the persister cell state, which is inscribed during colony–biofilm culture, in a wide variety of bacteria.

## Introduction

In response to environmental and physiological changes, or sometimes spontaneously, bacterial cells produce various types of specific subpopulations for better survival. Persister cells, or persisters, are one such subpopulation and were first discovered in *Staphylococcus* sp. (Bigger, 1944) and later also in other bacteria including *Escherichia coli* (Jayaraman, 2008; Lewis, 2010; Van den Bergh et al., 2017). Persisters exhibit temporary antibiotic-resistant phenotypes (usually multidrug resistance) and are therefore distinguishable from the permanent antibiotic resistance caused by genetic mutations or horizontal gene transfer. It is thought that the inherent toxin–antitoxin systems may induce a dormant cell state (Schuster and Bertram, 2013; Wood et al., 2013; Harms et al., 2016) that results in temporary antibiotic resistance because antibiotics generally work only on growing cells. Many toxin–antitoxin systems have been identified so far (Schuster and Bertram, 2013); for example, there are at least 10 toxin–antitoxin systems in *E. coli* (Yamaguchi and Inouye, 2011). However, a wide variety of mechanisms involved in the induction and regulation of persister cell formation have not yet been fully understood (Amato et al., 2014; Harms et al., 2016; Rahman et al., 2017; Cabral et al., 2018; Kawai et al., 2018).

Memory mechanisms in bacterial cells are known mainly in the field of bacterial immunity to foreign genetic materials. An early example is the restriction modification system (Luria, 1953; Wilson, 1991), and more recently, the CRISPR–Cas9 system (Jansen et al., 2002; Horvath and Barrangou, 2010). Both these bacterial mechanisms share a commonality with respect to their memorization of specific DNA sequences. Meanwhile, in eukaryotic cells, epigenetic memory mechanisms are used in many physiological processes (Allis et al., 2007). DNA methylation, histone modification, and RNA interference are typical of such memory mechanisms (Allis et al., 2007), which are involved in the memory of cell states, cell experience, or cell histories in addition to specific nucleic acid sequences (Allis et al., 2007).

Bacterial cells in biofilms are known to exhibit higher antibiotic resistance than their planktonic forms (Lewis, 2001; Stewart, 2015) and the relationship between this resistance and persister cell formation has been being studied (Lewis, 2001; Flemming et al., 2016). Although there are many reports on the enhancement by traditional liquid–solid biofilm culture (a biofilm formed at a liquid–solid interface) of persister cell formation in various bacteria (e.g., Lewis, 2010; Stewart, 2015), fewer have investigated the effect of colony–biofilm culture [a biofilm formed at an air–solid interface (Anderl et al., 2000; Carmen et al., 2004; Maeda et al., 2006)] (Nguyen et al., 2011; Amato and Brynildsen, 2014). Particularly, the long-term residual effect of biofilm culture on persister cell state after withdrawal from biofilm culture has never been investigated. In this study, we demonstrate the residual promoting effect of colony–biofilm culture on persister cell survival in *E. coli*. We also provide the first data to suggest the presence of a long-retention effect, or “memory effect,” of the persister cell state, which is inscribed during the colony–biofilm culture of *E. coli* and a wide variety of other bacteria.

## Materials and Methods

### Bacterial Strains and Materials

*Escherichia coli* strains: MG1655 (F^-^, *aaa, rph-1*) (Bachmann, 1996), W3110 (F^-^, *aaa, IN(rrnD-rrnE)1, rph-1*) (Bachmann, 1996), and BW25113 (F^-^, *rrnB, ΔlacZ4787, HsdR514, Δ(araBAD)567, Δ(rhaBAD)568, rph-1*) (Baba et al., 2006), and other bacterial strains (*Acinetobacter radioresistens*: NBRC102413, *Salmonella typhimurium*: NBRC14193, and *Staphylococcus epidermidis*: NBRC100911) were obtained from the NBRP (NIG, Mishima, Japan^1^). A *Bacillus subtilis* strain: ISW1214 (*hsrM1, leuA8, metB5, tet^s^*) (Ishiwa and Shibahara-Sone, 1986) was obtained from Takara Bio (Kyoto, Japan). Ampicillin (amp), amoxicillin (amo), tetracycline (tet), chloramphenicol (cam), streptomycin (str), ofloxacin (ofl), norfloxacin (nor), phosphate-buffered saline (PBS) tablets (1.47 mM potassium phosphate monobasic, 8.1 mM sodium phosphate bibasic, 2.7 mM potassium chloride, and 137 mM sodium chloride, pH 7.4 at 25°C) and Luria-Bertani (LB) powder (Luria-Bertani, Lennox) were obtained from Sigma (St. Louis, MO, United States). Distilled water (DNase- and RNase-free, molecular biology grade) and kanamycin (kan) were obtained from Invitrogen (Carlsbad, CA, United States). Agar powder (guaranteed-reagent grade) and other general reagents were obtained from Wako (Osaka, Japan).

### Cell Culture and Measurement of Persister Cell Formation

**FIGURE 1:**
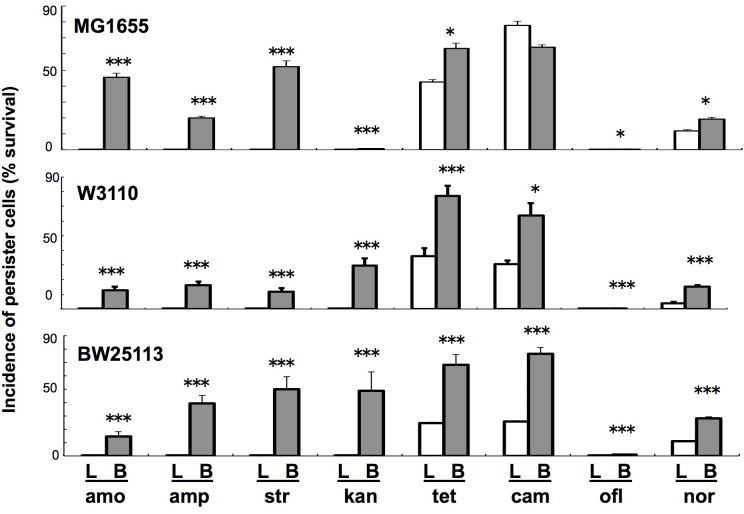
Comparison of persiter cell formation between liquid and colony–biofilm cultures in three *E. coli* strains with eight antibiotics. Three strains (MG1655, W3110, and BW25113) and eight antibiotics (amp, amo, str, kan, tet, cam, ofl, and nor; concentration given in Materials and Methods) were used. Data on the incidence of persister cells (% survival) are presented as means and S.D. (^∗^*t*-test: *P* < 0.05, *n* = 5; ^∗∗∗^*t*-test: *P* < 0.005, *n* = 5). Data are shown for cells grown in liquid (L: white bars) and colony–biofilm culture (B: gray bars).

**FIGURE 2:**
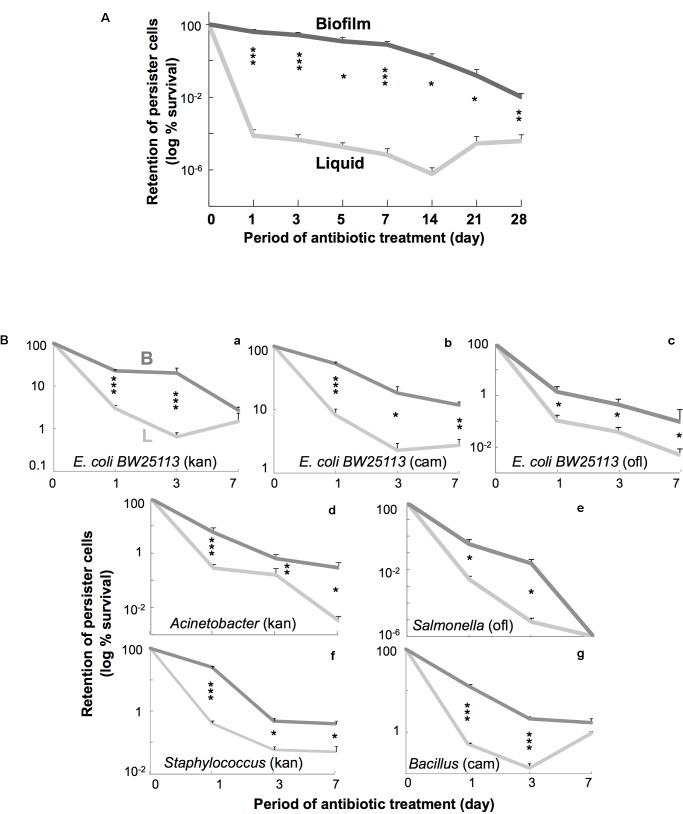
**(A)** Time course of persiter cell retention in *E. coli*. W3110 cells were incubated in fresh LB containing amp at 37°C up to 28 days. Data on retention of persister cells (log % survival) are presented as means and S.D. (^∗^*t*-test: *P* < 0.05, *n* = 5–7; ^∗∗^*t*-test: *P* < 0.01, *n* = 5–7; ^∗∗∗^*t*-test: *P* < 0.005, *n* = 5–7). Data are shown for cells grown in liquid (light gray line) and colony–biofilm culture (dark gray line). **(B)** Time course of persiter cell retention in several bacterial species. *E. coli* BW25113 **(a–c)** and several bacteria [*Salmonella Typhimurium* **(d)**, *Acinetobacter radioresistens* **(e)**, *Bacillus subtilis* **(f)**, and *Staphylococcus epidermidis* **(g)**] were used. Antibiotics [kan (40 μg/mL for *Acinetobacter* and *Staphylococcus*), cam (50 μg/mL for *Bacillus*), and ofl (5 μg/mL for *Salmonella*)] were chosen in pilot testing. Data on retention of persister cells (log % survival) are presented as means and S.D. (^∗^*t*-test: *P* < 0.05, *n* = 4; ^∗∗^*t*-test: *P* < 0.01, *n* = 4; ^∗∗∗^*t*-test: *P* < 0.005, *n* = 4). Data are shown for cells grown in liquid (light gray line) and colony biofilm culture (dark gray line).

Colony–biofilm and liquid cultures of *E. coli* cells were performed as previously described (Maeda et al., 2006). Essentially the same protocol was used for other bacteria, except that 30°C was used as the culture temperature for *Bacillus* and *Acinetobacter*. Briefly, cells were precultured in LB broth at 37°C for 16 h with shaking [150 rpm with a shaker (NTS-1300, EYELA, Tokyo, Japan)]. Their turbidities (OD_600_) were then measured to estimate total cell number for each culture, and cell densities were adjusted with fresh LB broth to 4 × 10^8^ cells/mL each. Aliquots (15 μL; total 6 × 10^6^ cells) of each precultured cell suspension were cultured in 3 mL of fresh LB broth with shaking [150 rpm with a shaker (NTS-1300, EYELA)] for liquid culture or on sterilized nylon membrane filters placed on LB agar for colony–biofilm culture at 37°C for 24 h. Biofilm cells were recovered and suspended in fresh LB broth. Turbidities (OD_600_) were again measured to estimate total cell number for each sample. A portion of each culture solution was spread onto LB agar to measure the initial number of colony-forming units (CFUs). Sample aliquots (each containing 3.75 × 10^8^ cells) were transferred to 0.2 mL microtubes, centrifuged, and resuspended in 150 μL of fresh LB broth containing an appropriate concentration of each antibiotic [amp: 75 μg/mL (**Figure 1**) and 200 μg/mL (**Figure 2A**), amo: 25 μg/mL, str: 75 μg/mL, tet: 75 μg/mL, kan: 75 μg/mL, cam: 100 μg/mL, ofl: 5 μg/mL, and nor: 5 μg/mL for *E. coli* strains]. The microtubes were then incubated with their lids closed while shaking [1260 rpm with a microplate shaker (MBR-022, TAITEC, Tokyo, Japan)] at 37°C for various periods. In this incubation, and based on data obtained from trial experiments, an O_2_-limiting condition (i.e., with tube lids closed) was utilized to maximize the differences in the results from colony–biofilm-cultured and liquid-cultured cells. Antibiotic-treated cells were collected by centrifugation, washed twice with fresh PBS, and resuspended in 150 μL of fresh LB broth. Turbidities (OD_600_) of aliquots of these suspensions were measured to estimate each total cell number. Suspensions were then serially diluted and spread onto antibiotic-free LB agar plates to measure the number of CFUs in each final sample. Incidences of persister cells were calculated as the survival rate of cells during the above incubation in the antibiotic-containing media, i.e., the ratio of the final CFU to the initial CFU.

## Results

### Colony–Biofilm Culture Promotes Persister Cell Formation in *E. coli*

We initially verified a previously reported phenomenon in *E. coli* that colony–biofilm culture can produce more number of persister cells than can liquid culture using a more fairly comparable experimental system, in which antibiotic treatment was performed under the same conditions using resuspended cells that had just been sampled from these two different growth conditions. Almost all combinations (23/24) of three laboratory strains and eight antibiotics resulted in significantly higher prevalence of persister cells in colony–biofilm culture than in the liquid culture (**Figure 1**), verifying that colony–biofilm culture promotes the development of persister cells. We confirmed that resistant cells could revert to being antibiotic-sensitive by subsequent culture in fresh antibiotic-free media, demonstrating that these were not mutants but rather persister cells with reversible resistance.

### Memory of Persister Cell State in *E. coli*

Then we tested the residual effects of the preceding culture conditions on persister cell survival during prolonged antibiotic treatment for periods of up to 4 weeks, although standard persister assays usually take only several hours (Lewis, 2010; Amato and Brynildsen, 2014; Stewart, 2015). **Figure 2A** shows the result of a time-course experiment of *E. coli* cell survival for 4 weeks in amp-containing fresh LB broth at 37°C after the initial liquid or colony–biofilm culture. The persister cells derived from colony–biofilm culture remained in much higher numbers than those derived from liquid culture for up to 28 days. This indicates that the persister cell state that developed in colony–biofilm culture could be maintained for at least 4 weeks after withdrawal from colony–biofilm culture—in other words, the persistence-promoting effect of the preceding colony–biofilm culture was “memorized” by the persister cells in suspension. Similar “memory” effects, at least for several days, were also observed when using other antibiotics such as kan, cm, and ofl (**Figures 2B,a–c**).

### Generality of the Memory Effect of Persister Cell State in a Wide Variety of Bacteria

Subsequently, we tested whether the persistence-promoting effect of colony–biofilm culture and its resulting memory effect also occur in several other bacterial species. **Figures 2B,d–g** show typical results from 7-day time-course experiments using two gram-negative bacteria (*Acinetobacter* and *Salmonella*) and two gram-positive bacteria (*Staphylococcus* and *Bacillus*). All four bacteria showed promoting and memory effects similar to *E. coli*, although the degrees of each in the various bacteria were somewhat different. We also confirmed the reversion of surviving cells to antibiotic sensitivity by subsequent culture in media lacking antibiotics. These results indicate that the promoting effect of the colony–biofilm culture on persister cell formation and the resulting memory effect are common in a wide variety of bacteria.

## Discussion

In this short report, we presented the following results: (1) *E. coli* colony–biofilm culture produces many more persister cells than does liquid culture; (2) *E. coli* persister cells from the colony–biofilm culture retained antibiotic resistance in higher numbers and for longer period (days to weeks) than those from liquid culture, despite withdrawal from biofilm growth conditions; and (3) these two phenomena also occurred not only in other gram-negative bacteria but also in gram-positive bacteria. The first result confirms previous observations where persistence was assessed in colony–biofilm cells *in situ* by direct antibiotic challenge (Nguyen et al., 2011; Amato and Brynildsen, 2014). However, our study is the first to directly test cells in suspension after their removal from the colony–biofilm culture. This protocol has the advantage of allowing for a pure persister assay without the possible influence of biofilm-specific confounding factors that may also convey antibiotic tolerance (Lewis, 2001; Stewart, 2015; Flemming et al., 2016).

In addition, and more importantly, the second and third results suggest both the existence and ubiquity of a “memory effect” for the persister cell state among a wide variety of bacterial species. Bacterial memory mechanisms relating to specific DNA sequences are already known (Wilson, 1991; Horvath and Barrangou, 2010). In contrast, our findings are the first to suggest the presence of a bacterial memory mechanism relating to a specific physiological cell state or experience. This may be a unique example of bacterial epigenetics (Casadesús and Low, 2006). It is noteworthy that this memory effect was maintained in a fresh nutrient-rich medium at 37°C, suggesting that the cells living in these conditions maintained their persister cell state high despite their metabolically or energetically active potential. We hypothesize that a molecular signal is inscribed into the persister cells during colony–biofilm culture and results in this memory effect, although it is possible that antibiotic-forced dormancy of cell functions may also participate in this phenomenon. As a possible molecular mechanism, we speculate that some of the toxin–antitoxin systems play a role in inducing dormancy in the colony–biofilm culture as well as maintaining dormancy after the culture and that another unknown mechanism may be switched on during colony–biofilm culture to fix the toxin–antitoxin balance. It is also possible that another persister-forming mechanism (Harms et al., 2016), such as efflux drug transporter systems (Rahman et al., 2017), participates in some step of this phenomenon to cause the memory effect in an unknown manner. To expand upon and clarify this interesting concept, further elucidation of this phenomenon is required.

Although the details of the physiological significance of this phenomenon are presently unknown, it is probable that it confers survival benefits to the bacteria in antibiotic-containing environments (Van den Bergh et al., 2017), and such persistent survival of bacterial cells may sometimes result in adverse effects on human health (Michiels et al., 2016; Fisher et al., 2017; Cabral et al., 2018). It is therefore important to further investigate the generality of this phenomenon and to determine whether other persistence-promoting stimuli can show a similar memory effect.

## Author Contributions

SMi, ES, and YKm contributed equally to this work. SMa, SMi, ES, and YKm conceived and designed the experiments. SMi, ES, YKm, YKn, and MM performed the experiments. SMa, SMi, ES, and YKm analyzed the data. SMa, SMi, ES, and YKm wrote the paper.

## Conflict of Interest Statement

The authors declare that the research was conducted in the absence of any commercial or financial relationships that could be construed as a potential conflict of interest.

## Footnotes

1 http://www.nite.go.jp/en/nbrc/cultures/nbrc/index.html
